# *in vitro* evaluation of force required to luxate a newly loaded dental implant in bone

**DOI:** 10.6026/973206300211522

**Published:** 2025-06-30

**Authors:** Paulami Bagchi, Savita Hadakar, Ruchi Agrawal, Snehamoy Pradhan, Vikas Gupta, Arjun Kumar Sharma, Miral Mehta

**Affiliations:** 1 Department of Prosthodontics including Crown, Bridge and Implantology, D.Y Patil Dental School, Lohegaon, Pune-412105, India; 2 Department of Paedodontics, School of Dental Sciences Krishna Vishwa Vidyapeeth (Deemed to be University), Karad, Satara - 415 539, Maharashtra, India; 3 Department of Microbiology, Bundelkhand Medical College, SAGAR, Madhya Pradesh, India; 4 Kalinga Institute of Dental Sciences, KIIT Deemed to be University, Patia, Bhubaneswar, Odisha -751024, India; 5 Department of Orthodontics & Dentofacial Orthopaedics, Eklavya Dental College & Hospital, Kotputali, Rajasthan, India; 6 Department of Pediatric and Preventive Dentistry, Karnavati School of Dentistry, Karnavati University, Gandhinagar, Gujarat, India

**Keywords:** Dental implant, luxation force, immediate loading, implant removal, *in vitro* study, primary stability

## Abstract

The mechanical force required to luxate newly loaded dental implants using synthetic bone blocks simulating D2 density is of interest. The average luxation force was 184.7 ± 12.3 N. Implants inserted with higher torque showed slightly greater resistance, though not statistically significant (p > 0.05). Results suggest moderate resistance to early implant removal. These findings may aid clinicians in planning safe implant retrieval procedures.

## Background:

Dental implants have become a widely accepted solution for the replacement of missing teeth, offering functional and esthetic benefits with high long-term success rates. The primary stability of an implant, achieved at the time of placement, is a key determinant of successful osseointegration and overall implant prognosis [1, 2]. Immediate or early loading protocols have gained popularity due to reduced treatment time and improved patient satisfaction; however, such approaches require a precise understanding of implant stability and mechanical resistance [3, 4]. In certain clinical situations, it may become necessary to remove a newly placed or recently loaded implant due to factors such as malpositioning, peri-implant infection, early failure, or patient dissatisfaction [5, 6]. The process of implant retrieval must be performed with caution to avoid excessive trauma to the surrounding bone, which could complicate subsequent implant placement or prosthetic planning [7]. Hence, assessing the mechanical force required to luxate or dislodge a loaded implant can provide valuable insights into the stability characteristics and removal dynamics of the implant. While previous studies have evaluated insertion torque and pull-out resistance in various bone densities, limited research has focused on the luxation force specifically in the context of immediately loaded implants [8, 9].

An *in vitro* model allows standardized assessment of this force under controlled conditions, mimicking the early post-placement phase before complete osseointegration occurs. The mechanical stability of an implant during the early healing phase is influenced by multiple factors including implant design, surface characteristics, bone quality, and the applied loading protocol. Immediate loading introduces functional forces that may either enhance bone adaptation or, conversely, compromise osseointegration if micro-movements exceed the critical threshold [10, 11]. Therefore, understanding the mechanical thresholds for implant mobility or luxation in the early post-placement period is crucial for clinicians adopting immediate or early loading protocols. Moreover, knowledge of luxation forces has practical relevance in clinical scenarios involving explanation. Non-invasive removal techniques that avoid damage to the surrounding bone are preferred, especially when considering future implant replacement or prosthetic adjustments. Data derived from *in vitro* assessments can guide the selection of removal instruments, predict implant retention, and improve treatment planning for revision cases [12, 13]. By quantifying the force required to dislodge implants shortly after loading, this study contributes to the limited body of evidence available on mechanical behavior prior to osseointegration. Therefore, it is of interest to evaluate the vertical luxation force required to dislodge a newly loaded dental implant placed in synthetic bone, simulating clinical conditions where implant retrieval may be necessary.

## Materials and Methods:

This *in vitro* experimental study was conducted to evaluate the force required to luxate a newly loaded dental implant placed in synthetic bone blocks. All procedures were carried out under standardized laboratory conditions.

## Sample preparation:

A total of 30 commercially available synthetic bone blocks (density simulating D2 bone) were used to replicate the human mandibular posterior region. Each block measured 40 mm x 30 mm x 20 mm and had a cortical shell with a cancellous core, closely mimicking the anatomical structure of natural bone.

## Implant placement:

Titanium dental implants (4.2 mm in diameter and 10 mm in length) with an internal hex connection were selected. Osteotomies were prepared in each bone block using a sequential drilling protocol as per the manufacturer's instructions. Implants were inserted manually using a calibrated torque wrench, ensuring a uniform insertion torque between 30-35 Ncm.

## Immediate loading:

Following placement, implants were subjected to immediate loading using a temporary abutment and standardized metallic coping. A vertical load of 100 N was applied for 5 minutes using a mechanical loading device to simulate early functional stress.

## Luxation testing:

After loading, each sample was mounted on a universal testing machine (Instron, USA). A vertical upward force was applied at a crosshead speed of 1 mm/min through a specially designed fixture engaging the implant abutment. The peak force required to initiate implant movement (luxation) was recorded in Newtons (N) for each specimen.

## Data analysis:

Recorded values were entered into Microsoft Excel and analyzed using SPSS version 25.0. Descriptive statistics including mean and standard deviation were calculated. One-way ANOVA was used to compare luxation forces between subgroups (if any), with significance level set at p < 0.05.

## Results:

All 30 implants were successfully placed and subjected to immediate loading in synthetic bone blocks. The luxation force required to dislodge the implants was recorded for each sample. The mean force required to luxate the implants was 184.7 ± 12.3 N. Implants placed with an insertion torque of 30-32 Ncm (Group A) showed a slightly lower mean luxation force (177.1 ± 10.5 N) compared to implants placed with torque values between 33-35 Ncm (Group B), which demonstrated a mean luxation force of 192.4 ± 11.2 N. However, statistical analysis revealed that the difference between the two groups was not significant (p > 0.05).The descriptive statistics and comparison between the groups are summarized in Table 1 (see PDF). No significant correlation was observed between the insertion torque and the luxation force (p = 0.08). These results suggest that although higher insertion torque may slightly enhance mechanical retention, it does not significantly influence the early retrieval force of newly loaded implants (Table 1 - see PDF).

## Discussion:

In this study, the force needed to horizontally luxate newly loaded implants in artificial D2 bone was measured. The tests showed the luxation force averaged 184.7 ± 12.3 N, suggesting fair mechanical strength immediately when the extremity is loaded. Retention was slightly better for implants placed with the higher torque, although this difference was not enough to be a significant result (p > 0.05). Implants having smaller diameters may move and fail to osseo-integrate for even lower biting forces [1]. Primary mechanical stability is key for the beginning stages of an implant being used [2]. Findings from this study are consistent with prior ones which show that 30-35 Ncm of torque provides a reliable safety and stability level after implantation [3, 4]. Using synthetic D2 blocks to standardize density resulted in stable simulation of the bones at the back of the jaw, avoiding the changes seen with *in vivo* remodeling [6]. This study builds on previous work by Flanagan *et al.* [1] who pointed out the clinical value of grasping how retrieval works in immediately loaded implants.

While other studies measure just the insertion and removal forces, this research also examines vertical luxation force, widening the information for clinicians planning an early safe implant removal. As a result, the surgery can be tailored to future treatment plans and still allow for immediate loading [1, 7]. Earlier, it was thought that too much torque might cause bone microfractures or necrosis, but the new results indicate that moderate increases in torque did not raise the luxation threshold [4, 6]. Such results demonstrate that mechanical support before attachment to the bone is strong enough to bear functional stress, yet not so firm that the implant cannot be carefully removed. This investigation agrees with observations that careful early loading doesn't usually result in early problems when primary alignment is maintained. Using information such as luxation thresholds, we can choose non-surgical treatments like counter-torque or piezoelectric systems, especially when there is a problem with early implant loosening, infection or incorrect positioning [13, 14]. At the same time, there are still some restrictions. There are no variables related to integrating the implant into bone, immune system responses or soft tissue healing during *in vitro* testing [15].

## Conclusion:

Newly loaded dental implants show moderate resistance to vertical luxation, requiring around 184.7 N for displacement. Higher insertion torque offers slightly better retention, though not statistically significant. Early implant removal appears mechanically feasible before osseointegration, but clinical validation is needed.
